# Supplementary material for: One-Step Solid-State Synthesis of Ni-Rich Cathode Materials for Lithium-Ion Batteries
Source: Materials (Basel). 2023 Apr 13;16(8):3079. doi: 10.3390/ma16083079 (PMC10142918; doi:10.3390/ma16083079)
Supplement: Supplementary file 1 [file materials-16-03079-s001.zip › materials-2322690-supplementary.pdf]

Supporting information for

One-Step Solid-State Synthesis of Ni-Rich Cathode Materials for Lithium-Ion Batteries

Lifan Wang [1,2], Qinling Shi [1,2], Chun Zhan [1,2,*] and Guicheng Liu [3,4,*]

[1] State Key Laboratory of Advanced Metallurgy, School of Metallurgical and Ecological Engineering, University of Science and Technology Beijing, Beijing 100083, China
[2] Department of Energy Storage Science and Engineering, School of Metallurgical and Ecological Engineering, University of Science and Technology Beijing, Beijing 100083, China
[3] School of Energy Power and Mechanical Engineering, North China Electric Power University, Beijing 102206, China
[4] Department of Physics, Dongguk University, Seoul 04620, Republic of Korea
* Correspondence: zhanchun@ustb.edu.cn (C.Z.); log67@163.com or gcliu@ncepu.edu.cn (G.L.)

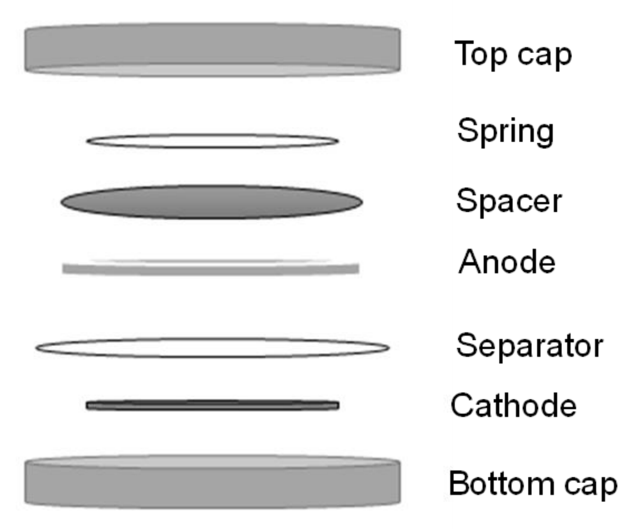

Figure S1. Assembly diagram of 2025 coin-type cell.

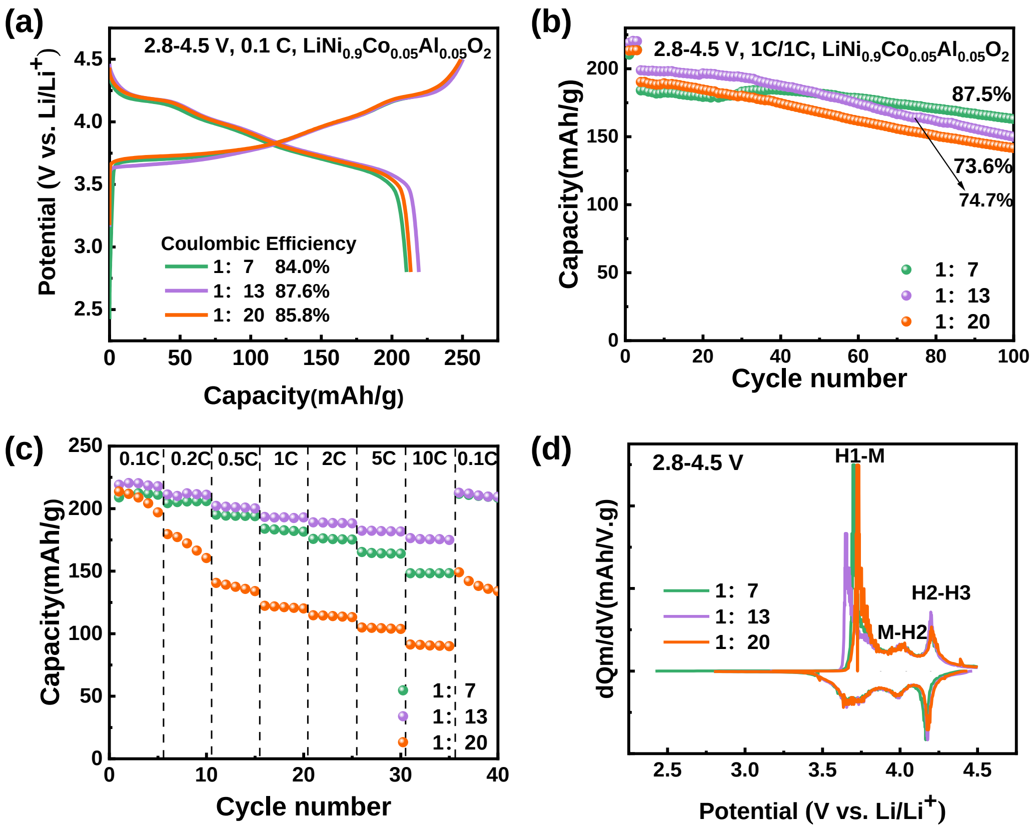

Figure S2. Electrochemical performance of NCA made by three different ball material ratios in the voltage range of 2.8-4.5 V.

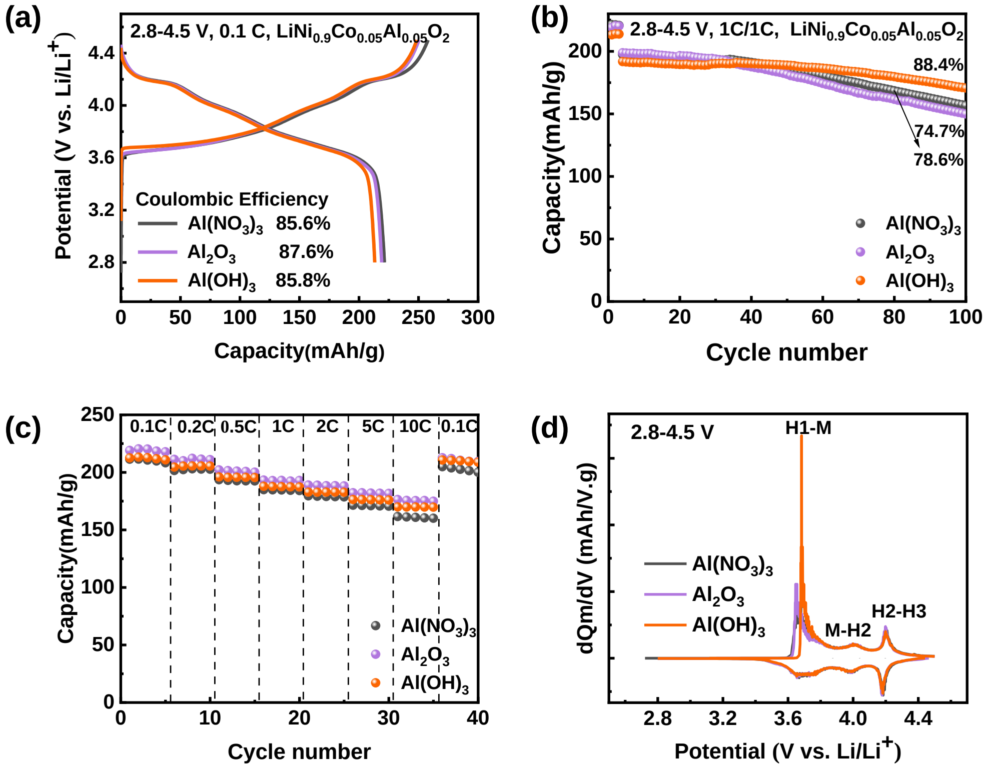

Figure S3. Electrochemical performance of NCA made by three different aluminum precursors in the voltage range of 2.8-4.5 V.

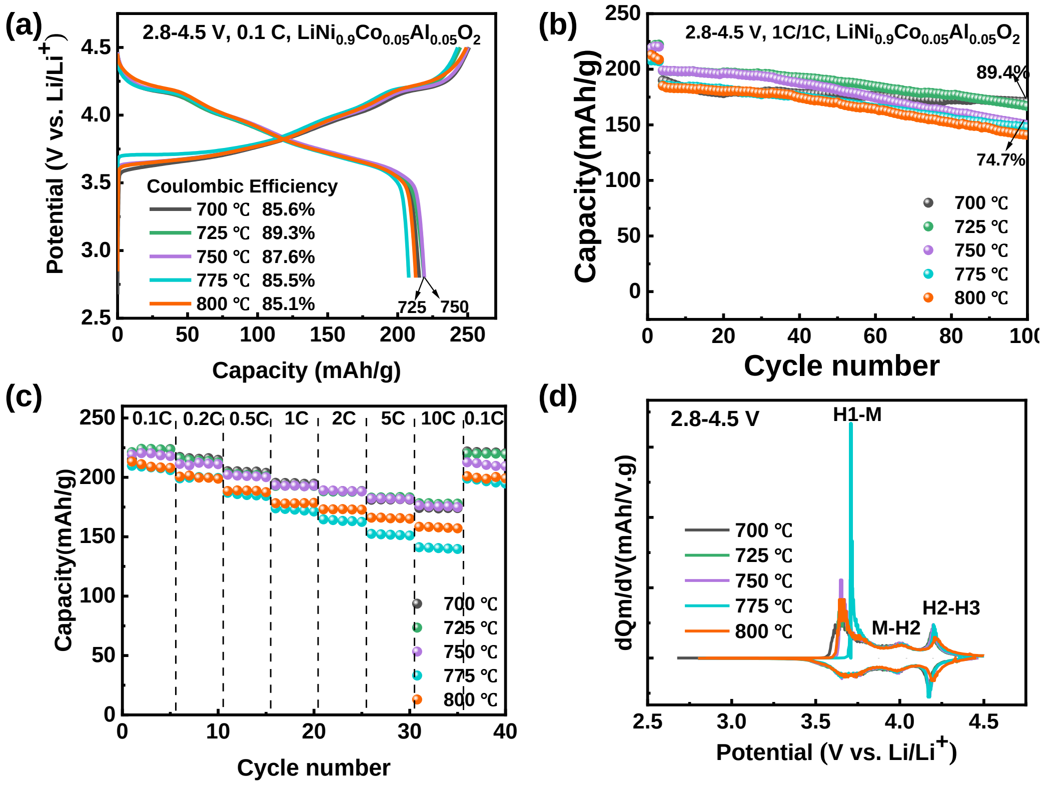

Figure S4. Electrochemical performance of NCA made by five different calcination temperatures in the voltage range of 2.8-4.5V.

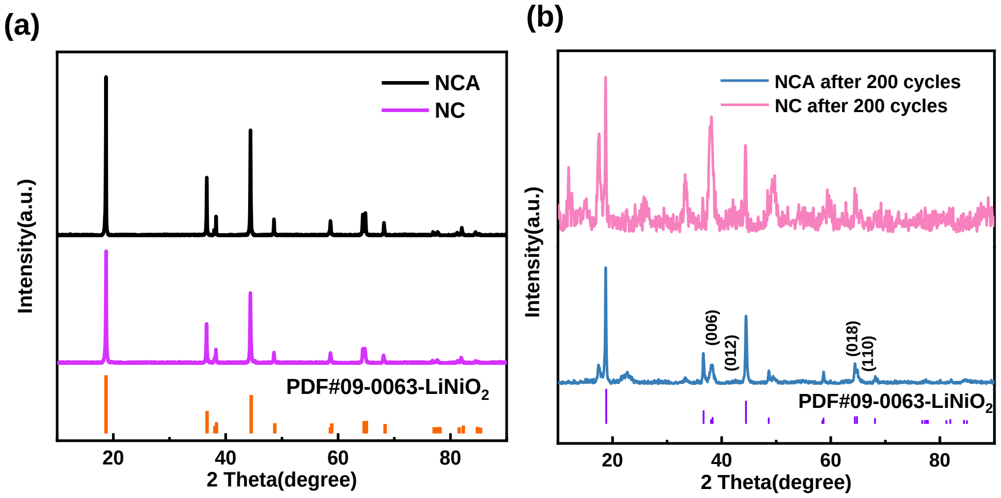

Figure S5. XRD patterns of $LiNi_{0.9}Co_{0.1}O_2$ and $LiNi_{0.9}Co_{0.05}Al_{0.05}O_2$ cathode materials before **a** and after cycling **b.**

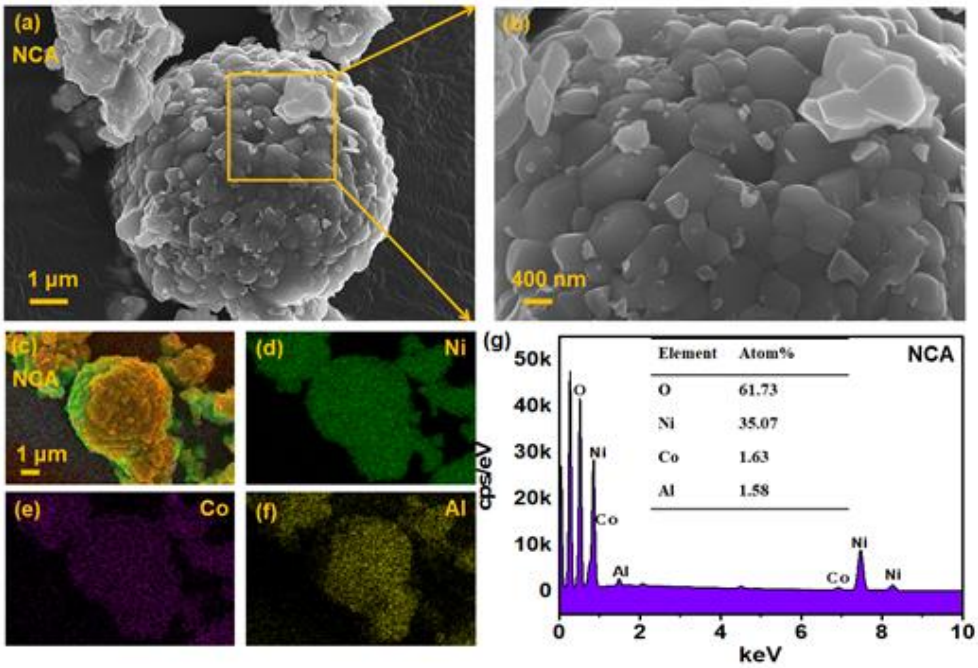

Figure S6. a, b SEM images of the NCA cathode materials; **c-f** EDS mapping with SEM measurement images of the NCA cathode materials; **g** The content of the detected elements on the NCA particles.

Table S1. Charge-discharge data of NCA at different aluminum precursors.

Voltage range	Aluminum precursor	1st discharge capacity (mAh/g)	4th discharge capacity (mAh/g)	100th discharge capacity (mAh/g)	Capacity retention (%)
2.8-4.3 V	$Al(NO)_3$	201.87	178.18	157.77	88.5
	Al_2O_3	206.11	185.55	163.06	87.8
	$Al(OH)_3$	200.97	178.70	171.98	96.2
2.8-4.5 V	$Al(NO)_3$	221.34	197.40	155.11	78.5
	Al_2O_3	219.04	198.82	148.29	74.5
	$Al(OH)_3$	213.22	191.80	169.34	88.2

Table S2. Rate performance data of NCA at aluminum precursors.

Voltage range	Current density	Discharge capacities at different aluminum precursors (mAh/g)		
		$Al(NO_3)_3$	Al_2O_3	$Al(OH)_3$
2.8-4.3 V	0.1 C	203.59	204.88	199.16
	0.2 C	196.47	200.61	192.06
	0.5 C	187.83	192.63	184.77
	1 C	176.78	180.46	175.66
	2 C	171.65	175.13	172.16
	5 C	163.42	168.25	165.79
	10 C	156.19	165.68	159.87
	0.1 C	202.72	205.27	202.12
2.8-4.5 V	0.1 C	211.43	219.06	212.57
	0.2 C	201.59	211.28	204.53
	0.5 C	193.58	202.18	195.97
	1 C	184.97	193.35	187.86
	2 C	179.57	189.02	182.81
	5 C	171.47	182.24	176.42
	10 C	161.64	176.38	169.94
	0.1 C	204.94	212.70	210.76

Table S3. Charge-discharge data of NCA at different ball material ratios.

Voltage range	Ball material ratio	1st discharge capacity (mAh/g)	4th discharge capacity (mAh/g)	100th discharge capacity (mAh/g)	Capacity retention (%)
2.8-4.3 V	1:7	189.36	165.02	161.23	97.7
	1:13	206.63	185.79	162.30	87.8
	1:20	198.93	172.92	141.76	81.9
2.8-4.5 V	1:7	210.32	184.00	161.84	87.9
	1:13	219.04	198.82	148.29	74.5
	1:20	213.35	190.21	140.38	73.8

Table S4. Rate performance data of NCA at different ball material ratios.

Voltage range	Current density	Discharge capacities at different ball material ratios (mAh/g)		
		**1:7**	**1:13**	**1:20**
2.8-4.3 V	0.1 C	190.65	203.75	202.20
	0.2 C	189.38	200.02	194.75
	0.5 C	182.69	192.88	186.70
	1 C	174.14	182.97	176.44
	2 C	171.80	178.19	172.78
	5 C	165.42	172.31	165.34
	10 C	159.13	169.56	158.94
	0.1 C	204.29	205.33	202.47
2.8-4.5 V	0.1 C	208.94	219.06	213.68
	0.2 C	204.35	211.28	179.61
	0.5 C	195.11	202.18	140.63
	1 C	183.93	193.35	122.30
	2 C	175.75	189.02	114.67
	5 C	165.31	182.24	104.94
	10 C	148.33	176.38	91.34
	0.1 C	211.75	212.70	149.07

Table S5. Charge-discharge data of NCA at different temperatures.

Voltage range	Temperature	1st discharge capacity (mAh/g)	4th discharge capacity (mAh/g)	100th discharge capacity (mAh/g)	Capacity retention (%)
2.8-4.3 V	700°C	204.57	177.36	172.30	97.1
	725°C	204.85	182.09	169.32	92.9
	750°C	208.73	189.83	164.82	86.8
	775°C	198.66	173.03	152.26	87.9
	800°C	197.64	164.10	144.28	87.9
2.8-4.5 V	700°C	215.47	189.94	169.86	89.4
	725°C	218.87	198.65	165.20	83.1
	750°C	219.04	198.82	148.29	74.5
	775°C	207.91	185.59	146.66	79.0
	800°C	212.90	185.11	140.54	75.9

Table S6. Rate performance data of NCA at different temperatures.

Voltage range	Current density	Discharge capacities at different temperatures(mAh/g)				
		**700°C**	**725°C**	**750°C**	**775°C**	**800°C**
2.8-4.3 V	0.1 C	207.83	207.33	204.88	196.74	195.79
	0.2 C	202.32	204.98	200.61	189.72	185.75
	0.5 C	191.62	193.77	192.63	183.03	175.60
	1 C	182.89	183.56	180.46	174.27	164.36
	2 C	179.07	181.75	175.13	172.44	162.10
	5 C	172.91	176.48	168.25	165.66	155.44
	10 C	168.65	172.32	165.68	156.85	150.76
	0.1 C	217.86	216.64	205.27	197.54	192.03
2.8-4.5 V	0.1 C	215.26	221.18	219.06	209.72	213.26
	0.2 C	217.16	216.23	211.28	199.11	200.60
	0.5 C	205.14	203.26	202.18	186.99	188.46
	1 C	195.35	192.72	193.35	173.92	178.20
	2 C	188.77	188.21	189.02	164.61	173.07
	5 C	181.14	182.91	182.24	152.40	165.99
	10 C	174.20	178.20	176.38	141.18	158.39
	0.1 C	221.79	220.18	212.70	198.91	200.97

Table S7. Lattice parameters obtained by Rietveld refinement of NC and NCA cathode materials.

Samples	a	c	c/a	(003)peak areas	(104)peak areas	I(003)/I(104)
NC	2.876	14.194	4.935	143784	111817	1.28
NCA	2.873	14.207	4.945	218530	166595	1.31

Table S8. Comparison on the initial discharge capacity and capacity retention (%) after 100 cycles.

Samples	Methodes	Initial discharge capacity (mAh/g)	Capacity retention(%) after 100 cycles	Referenc e
LiNi0.8Co0.1Mn0.	Sol-gel	200.1	71.0	[1]
LiNi0.8Co0.1Mn0.	Co-	192.2	67.7	[1]
LiNi0.6Mn0.2Co0.	Solid-state	162.0	80 (90 cycles)	[2]
LiNi0.9Co0.05Al0.	Co-	214.0	89.7 (25	[3]
LiNi0.975Mg0.025	Co-	225.0	91.2 (50	[4]
LiNi0.8Co0.1Mn0.	Hydrothermal	213.1	67.9	[5]
LiNi0.5Co0.2Mn0.	Sol-gel	150.2	77.7	[5]
LiNi0.6Co0.2Mn0.	Hydrothermal	208.3	70.1	[6]
LiNi0.8Co0.1Mn0.	Solid-state	195.2	69.1	[7]
This work	Solid-state	204.5	97.2	

Table S9. The values of R_{sf} and R_{ct} for cycled electrodes.

**Samples**		**NC**	**NCA**
3rd cycle	$R_s(\Omega)$	7.6	2.3
	$R_{sf1}(\Omega)$	32.5	48.5
	$R_{ct}(\Omega)$	6.8	65.2
200th cycle	$R_s(\Omega)$	7.4	5.6
	$R_{sf1}(\Omega)$	69.1	55.7
	$R_{sf2}(\Omega)$	192.5	131.7
	$R_{ct}(\Omega)$	97.0	68.2

Reference

[1] Lu, H.; Zhou, H.; Svensson, A. M.; Fossdal, A.; Sheridan, E.; Lu, S.; Vullum-Bruer, F., High capacity $Li[Ni_{0.8}Co_{0.1}Mn_{0.1}]O_2$ synthesized by sol–gel and co-precipitation methods as cathode materials for lithium-ion batteries. *Solid State Ionics* 2013, (249), 105-111.

[2] Zheng, L.; J. Craig Bennett; Obrovac, M. N., All-Dry Synthesis of Single Crystal NMC Cathode Materials for Li-Ion Batteries. *Journal of The Electrochemical Society* 2020, *167*, 130536.

[3] Li, J.; Zhang, N.; Li, H.; Liu, A.; Wang, Y.; Yin, S.; Wu, H.; Dahn, J. R., Impact of the Synthesis Conditions on the Performance of $LiNixCoyAlzO_2$ with High Ni and Low Co Content. *Journal of The Electrochemical Society* 2019, *165* (14), A3544-3577.

[4] Liu, A.; Zhang, N.; Stark, J. E.; Arab, P.; Li, H.; Dahn, J. R., Synthesis of Co-Free Ni-Rich Single Crystal Positive Electrode Materials for Lithium Ion Batteries: Part I. Two-Step Lithiation Method for Al- or Mg-Doped $LiNiO_2$. *Journal of The Electrochemical Society* 2021, (168), 040531.

[5] Cui, J.; Ding, X.; Luo, D.; Xie, H.; Zhang, Z.; Zhang, B.; Tan, F.; Liu, C.; Lin, Z., Effect of Cationic Uniformity in Precursors on Li/Ni Mixing of Ni-Rich Layered Cathodes. *Energy Fuels* 2021, *35*, 1842-1850.

[6] Jiang, M.; Zhang, Q.; Wu, X.; Chen, Z.; Danilov, D. L.; Eichel, R.-A.; Notten, P. H. L., Synthesis of Ni-Rich Layered-Oxide Nanomaterials with Enhanced Li-Ion Diffusion Pathways as High-Rate Cathodes for Li-Ion Batteries. *ACS Applied Energy Materials* 2020, *3*, 6583-6590.

[7] Nam, G.; Hwang, J.; Kang, D.; Oh, S.; Chae, S.; Yoon, M.; Ko, M., Mechanical densification synthesis of single-crystalline Ni-rich cathode for high-energy lithium-ion batteries. *Journal of Energy Chemistry* 2023, *79*, 562-568.
